# Poster Session II - A200 THE PREVALENCE OF EOSINOPHILIC ESOPHAGITIS AMONG PATIENTS WITH CELIAC DISEASE: A SYSTEMATIC REVIEW AND META-ANALYSIS

**DOI:** 10.1093/jcag/gwaf042.200

**Published:** 2026-02-13

**Authors:** K Kecskemeti, J Ko, R Yanofsky

**Affiliations:** Faculty of Medicine, University of Toronto, Toronto, ON, Canada; Faculty of Medicine, University of Toronto, Toronto, ON, Canada; Faculty of Medicine, University of Toronto, Toronto, ON, Canada

## Abstract

**Background:**

Eosinophilic esophagitis (EoE) and celiac disease (CD) are autoimmune disorders involving the gastrointestinal tract with increasing prevalence. However, the association and prevalence of EoE in patients with CD remains unclear.

**Aims:**

We aimed to conduct a systematic review and meta-analysis regarding the prevelence of EoE and CD given the recent publication of multiple new population and cohort based studies.

**Methods:**

The MeSH terms “Eosinophilic Esophagitis” and “Celiac Disease” were used on PubMed to search for articles from database inception to May 2025. Two independent reviewers screened titles and abstracts, with a full-text review conducted for studies directly comparing EoE and CD. The primary outcome entailed the evaluation of an association between EoE and CD. Secondary outcomes included evaluation of shared pathophysiology and treatment of the conditions. A meta-analysis of the prevalence of EoE in patients with CD was performed using all applicable population-based studies with control group and single cohort studies. Statistical analysis was performed using Microsoft Excel Meta-Analysis workbook from the Erasmus Research Institute.

**Results:**

In total, 136 articles were identified, and 39 studies underwent full-text review as these directly compared EoE and CD. 12 studies met the inclusion criteria for the meta-analysis: 5 comparative and 7 single-cohort studies. Across the five comparative studies, the pooled odds ratio for concurrent EoE and CD was 3.07 (95% CI 0.60–13.71; I^2^ = 98.9%), suggesting a trend toward increased risk, although with high heterogeneity. Among the seven single-cohort studies, the pooled prevalence of EoE among patients with CD was 3.8% (95% CI 1.4–7.3%; I^2^= 90.5%) under a random-effects model using the Freeman–Tukey double-arcsine transformation. This rate is much higher than the prevalence of EoE in the general population which is estimated at 0.1%.

In terms of secondary outcomes: proposed pathophysiologic mechanisms linking EoE and CD include impaired gut membrane permeability and global regulatory T-cell dysfunction. Other inflammatory mediators such as IL-15 and thymic stromal lymphopoietin have been increasingly implicated in both conditions and may serve as potential therapeutic targets in the future. A retrospective study found no association between EoE and HLA-DQ2 and DQ8, alleles predisposing to CD, suggesting an unlikely genetic association between the two conditions. Currently, only a small subset of patients with EoE and CD achieve full clinical remission with gluten-free diets, while many report remission with an elemental diet.

**Conclusions:**

This meta-analysis demonstrates an increased prevalence of EoE among patients with CD when compared to the general population. Proposed mechanisms linking the conditions may include epithelial barrier dysfunction and aberrant cytokine signaling.

**Funding Agencies:**

NoneNil

